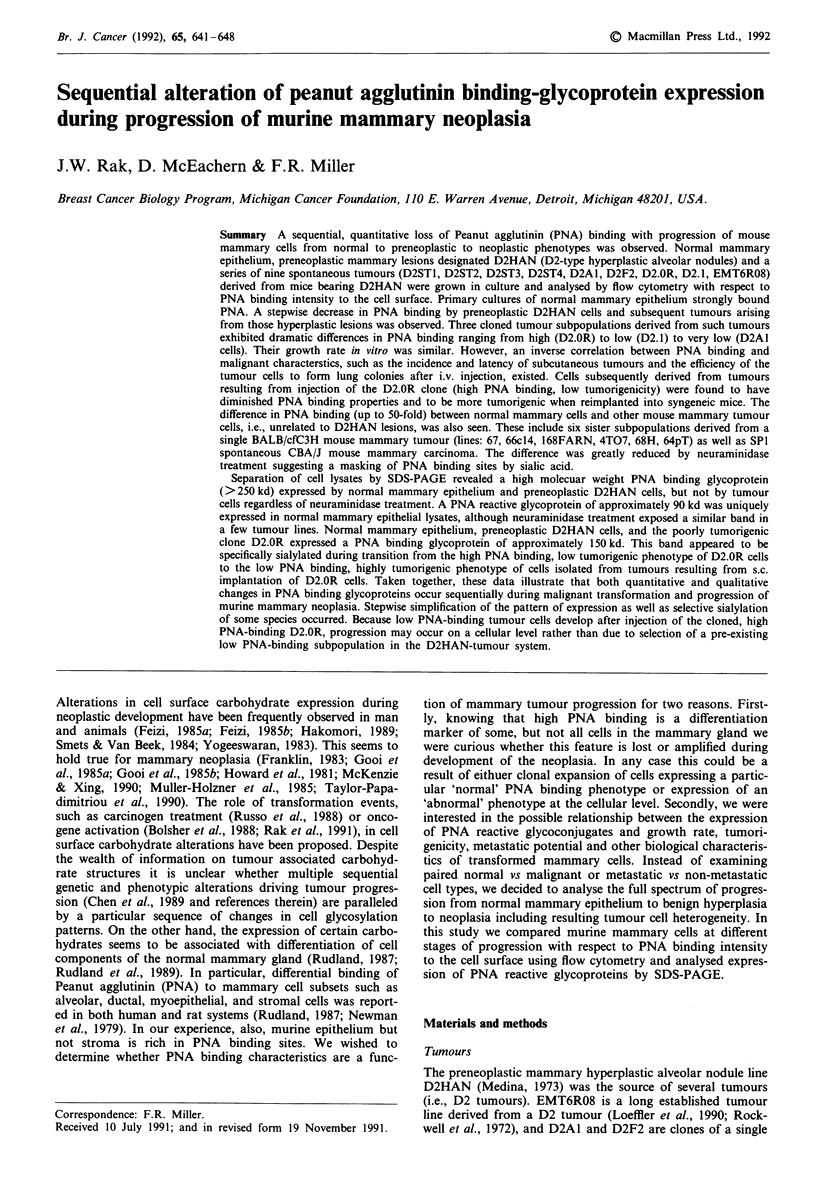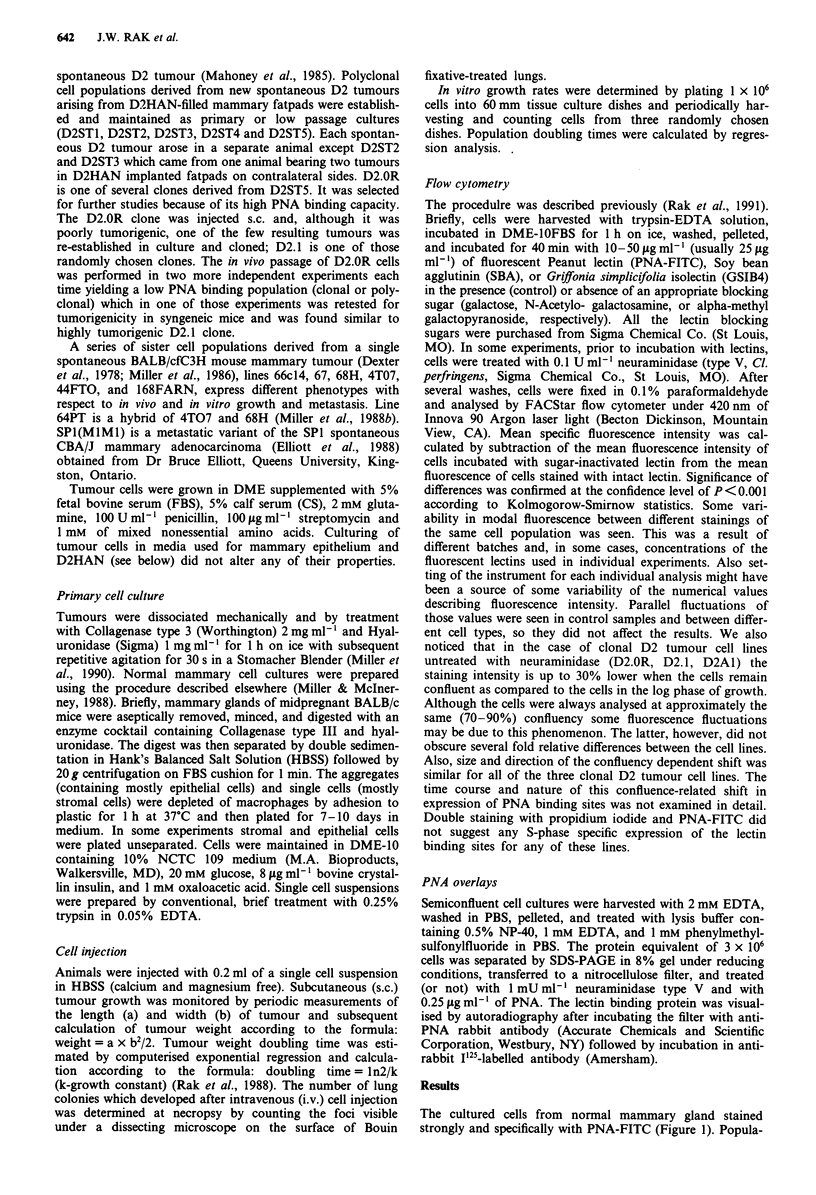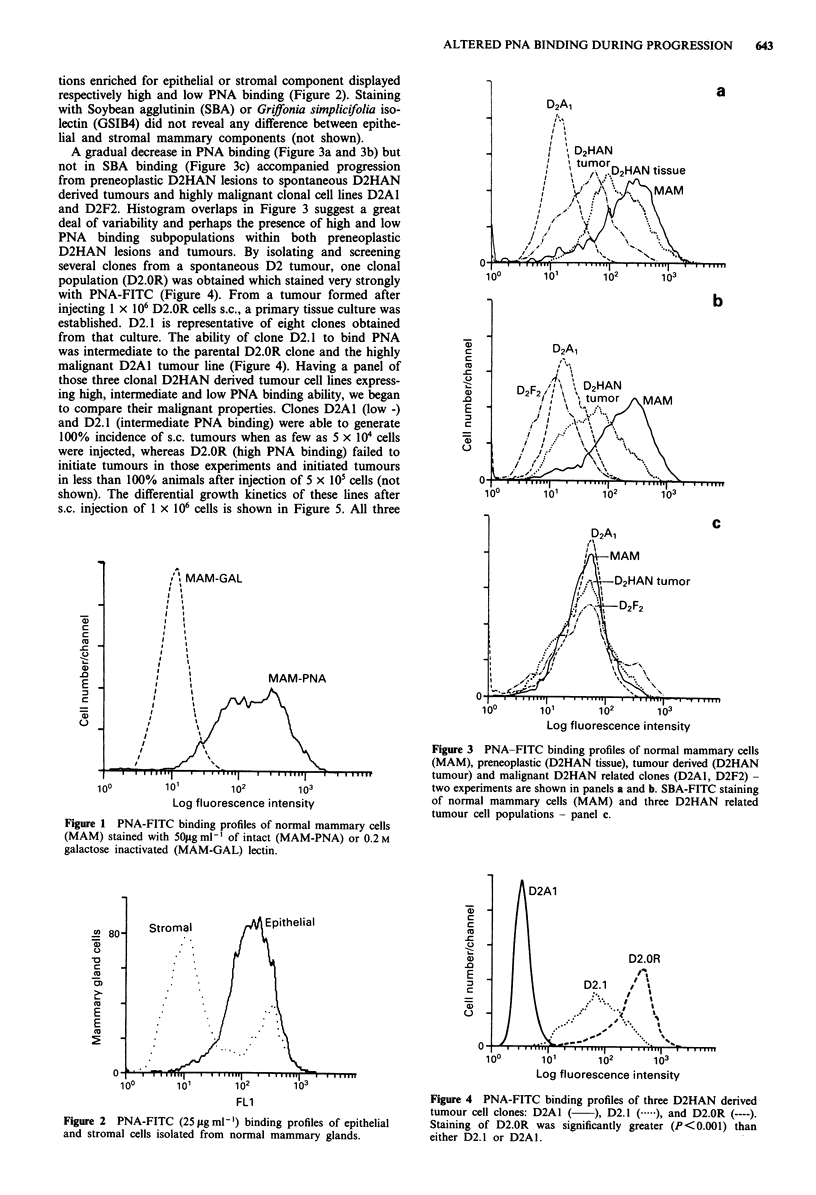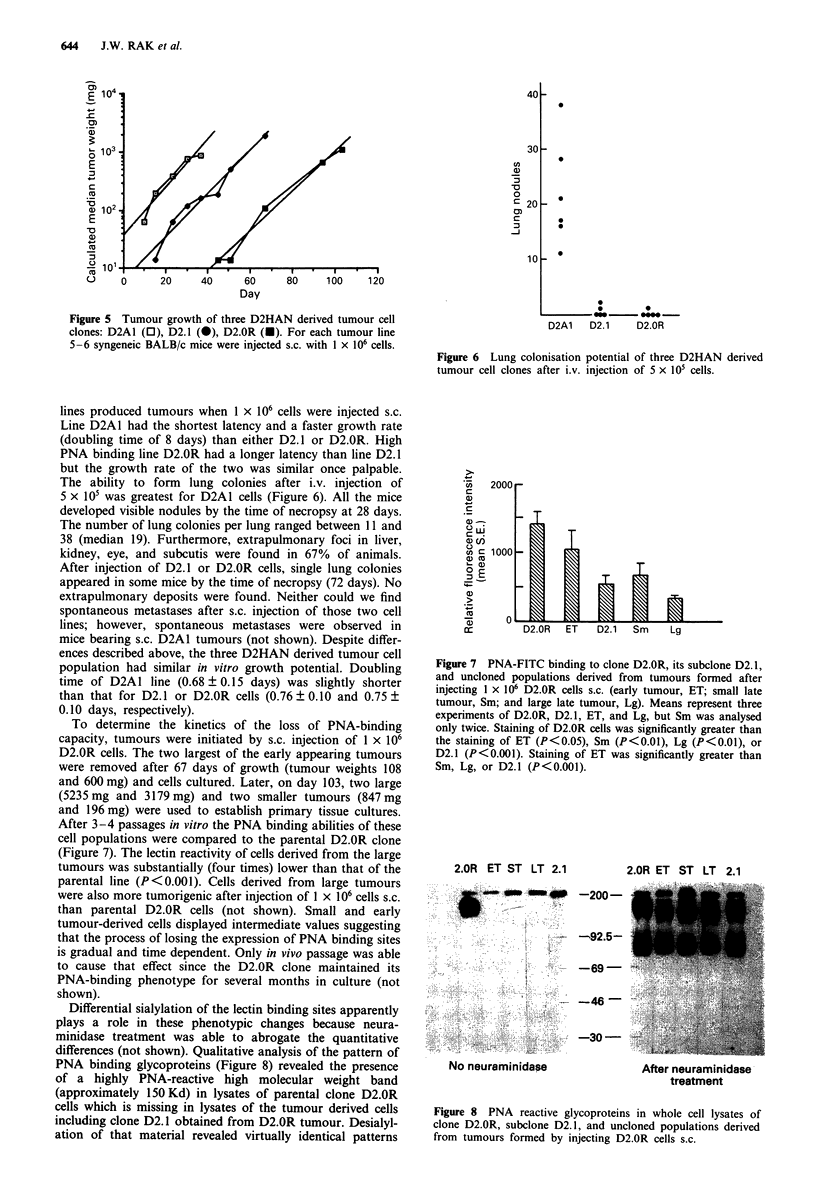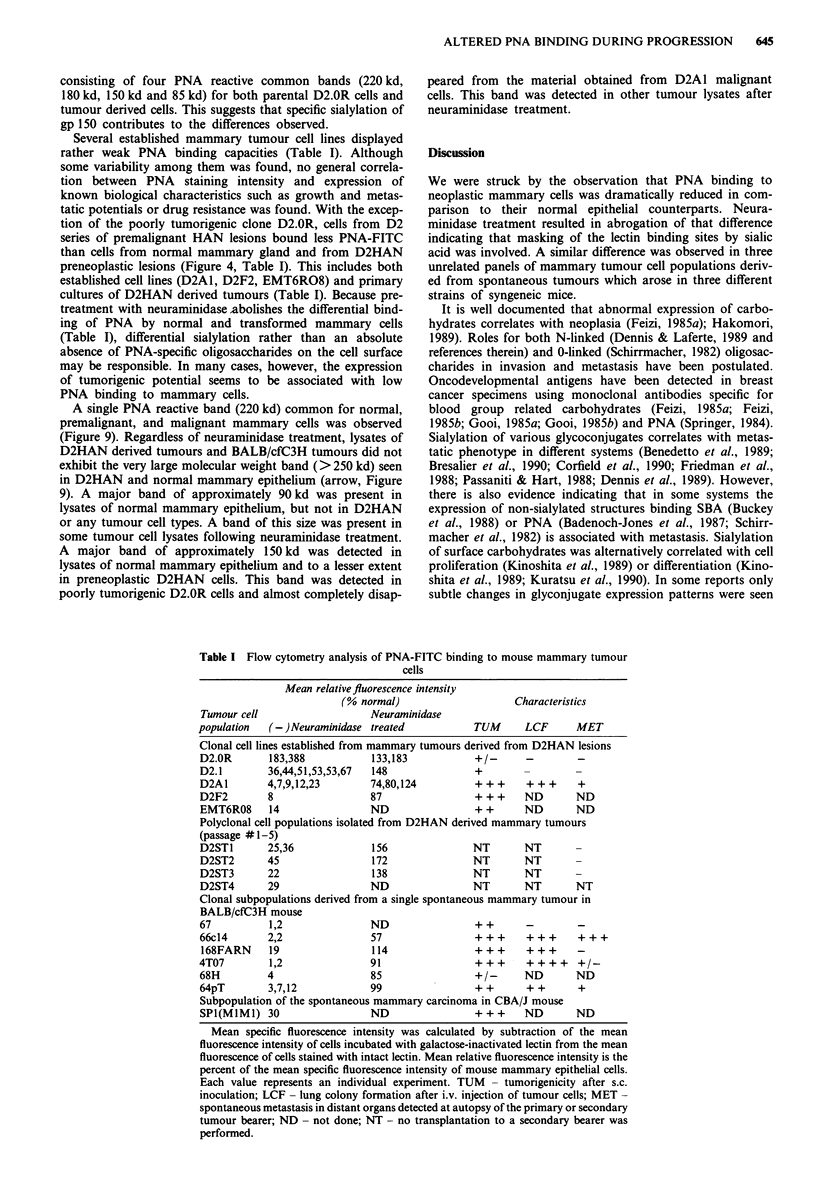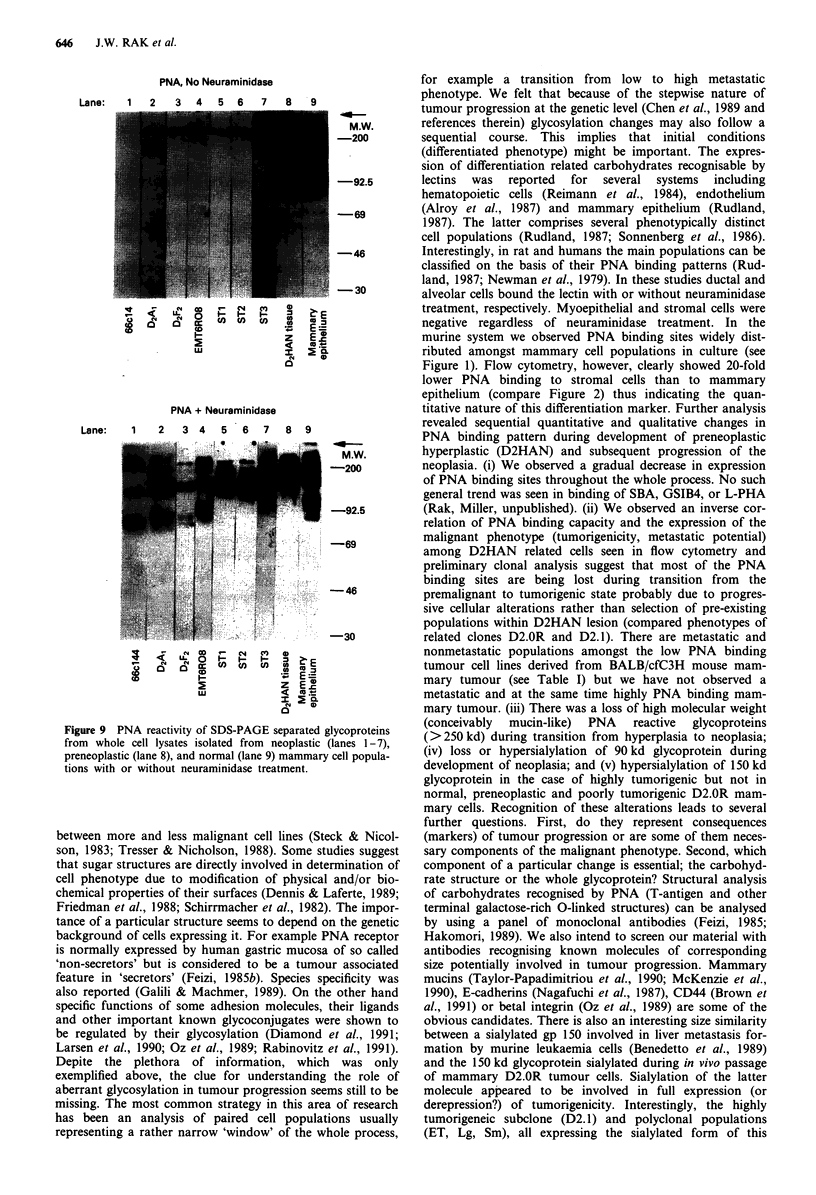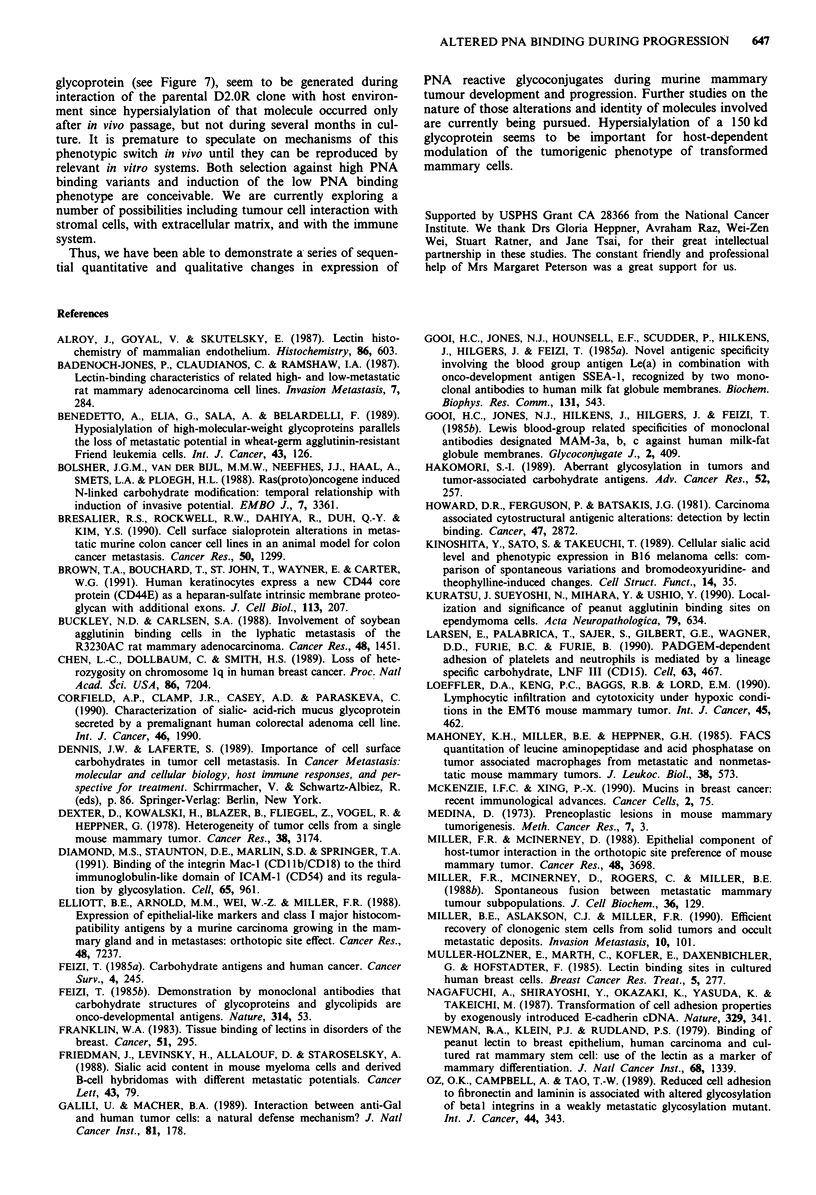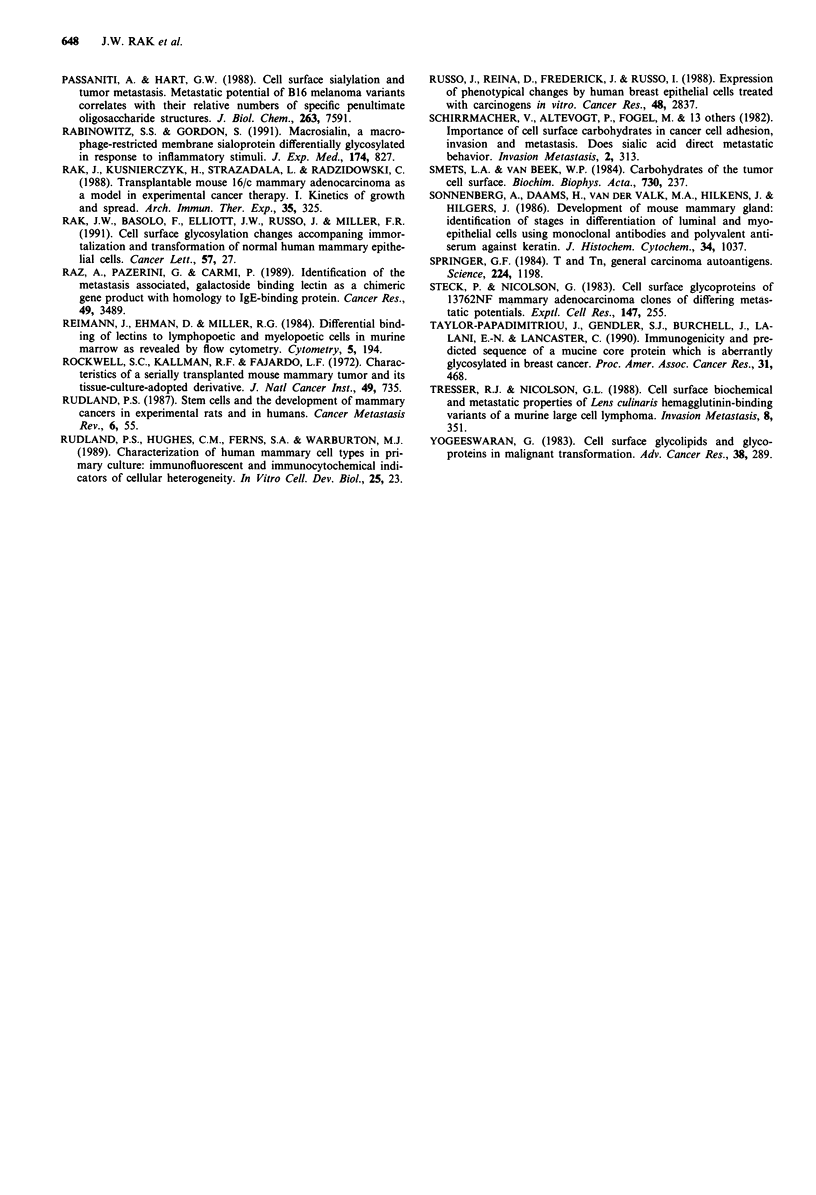# Sequential alteration of peanut agglutinin binding-glycoprotein expression during progression of murine mammary neoplasia.

**DOI:** 10.1038/bjc.1992.138

**Published:** 1992-05

**Authors:** J. W. Rak, D. McEachern, F. R. Miller

**Affiliations:** Breast Cancer Biology Program, Michigan Cancer Foundation, Detroit 48201.

## Abstract

**Images:**


Br. J. Cancer (1992), 65, 641–648 © Macmillan Press Ltd., 1992

### Sequential alteration of peanut agglutinin binding-glycoprotein expression during progression of murine mammary neoplasia

J.W. Rak, D. McEachern & F.R. Miller

*Breast Cancer Biology Program, Michigan Cancer Foundation, 110 E. Warren Avenue, Detroit, Michigan 48201, USA.*

**Summary** A sequential, quantitative loss of Peanut agglutinin (PNA) binding with progression of mouse mammary cells from normal to preneoplastic to neoplastic phenotypes was observed. Normal mammary epithelium, preneoplastic mammary lesions designated D2HAN (D2-type hyperplastic alveolar nodules) and a series of nine spontaneous tumours (D2ST1, D2ST2, D2ST3, D2ST4, D2A1, D2F2, D2.0R, D2.1, EMT6R08) derived from mice bearing D2HAN were grown in culture and analysed by flow cytometry with respect to PNA binding intensity to the cell surface. Primary cultures of normal mammary epithelium strongly bound PNA. A stepwise decrease in PNA binding by preneoplastic D2HAN cells and subsequent tumours arising from those hyperplastic lesions was observed. Three cloned tumour subpopulations derived from such tumours exhibited dramatic differences in PNA binding ranging from high (D2.0R) to low (D2.1) to very low (D2A1 cells). Their growth rate *in vitro* was similar. However, an inverse correlation between PNA binding and malignant characterstics, such as the incidence and latency of subcutaneous tumours and the efficiency of the tumour cells to form lung colonies after i.v. injection, existed. Cells subsequently derived from tumours resulting from injection of the D2.0R clone (high PNA binding, low tumorigenicity) were found to have diminished PNA binding properties and to be more tumorigenic when reimplanted into syngeneic mice. The difference in PNA binding (up to 50-fold) between normal mammary cells and other mouse mammary tumour cells, i.e., unrelated to D2HAN lesions, was also seen. These include six sister subpopulations derived from a single BALB/cfC3H mouse mammary tumour (lines: 67, 66c14, 168FARN, 4TO7, 68H, 64pT) as well as SP1 spontaneous CBA/J mouse mammary carcinoma. The difference was greatly reduced by neuraminidase treatment suggesting a masking of PNA binding sites by sialic acid.

Separation of cell lysates by SDS-PAGE revealed a high molecuar weight PNA binding glycoprotein (>250 kd) expressed by normal mammary epithelium and preneoplastic D2HAN cells, but not by tumour cells regardless of neuraminidase treatment. A PNA reactive glycoprotein of approximately 90 kd was uniquely expressed in normal mammary epithelial lysates, although neuraminidase treatment exposed a similar band in a few tumour lines. Normal mammary epithelium, preneoplastic D2HAN cells, and the poorly tumorigenic clone D2.0R expressed a PNA binding glycoprotein of approximately 150 kd. This band appeared to be specifically sialylated during transition from the high PNA binding, low tumorigenic phenotype of D2.0R cells to the low PNA binding, highly tumorigenic phenotype of cells isolated from tumours resulting from s.c. implantation of D2.0R cells. Taken together, these data illustrate that both quantitative and qualitative changes in PNA binding glycoproteins occur sequentially during malignant transformation and progression of murine mammary neoplasia. Stepwise simplification of the pattern of expression as well as selective sialylation of some species occurred. Because low PNA-binding tumour cells develop after injection of the cloned, high PNA-binding D2.0R, progression may occur on a cellular level rather than due to selection of a pre-existing low PNA-binding subpopulation in the D2HAN-tumour system.

Alterations in cell surface carbohydrate expression during neoplastic development have been frequently observed in man and animals (Feizi, 1985*a*; Feizi, 1985*b*; Hakomori, 1989; Smets & Van Beek, 1984; Yogeeswaran, 1983). This seems to hold true for mammary neoplasia (Franklin, 1983; Gooi *et al.*, 1985*a*; Gooi *et al.*, 1985*b*; Howard *et al.*, 1981; McKenzie & Xing, 1990; Muller-Holzner *et al.*, 1985; Taylor-Papadimitriou *et al.*, 1990). The role of transformation events, such as carcinogen treatment (Russo *et al.*, 1988) or oncogene activation (Bolsher *et al.*, 1988; Rak *et al.*, 1991), in cell surface carbohydrate alterations have been proposed. Despite the wealth of information on tumour associated carbohydrate structures it is unclear whether multiple sequential genetic and phenotypic alterations driving tumour progression (Chen *et al.*, 1989 and references therein) are paralleled by a particular sequence of changes in cell glycosylation patterns. On the other hand, the expression of certain carbohydrates seems to be associated with differentiation of cell components of the normal mammary gland (Rudland, 1987; Rudland *et al.*, 1989). In particular, differential binding of Peanut agglutinin (PNA) to mammary cell subsets such as alveolar, ductal, myoepithelial, and stromal cells was reported in both human and rat systems (Rudland, 1987; Newman *et al.*, 1979). In our experience, also, murine epithelium but not stroma is rich in PNA binding sites. We wished to determine whether PNA binding characteristics are a function of mammary tumour progression for two reasons. Firstly, knowing that high PNA binding is a differentiation marker of some, but not all cells in the mammary gland we were curious whether this feature is lost or amplified during development of the neoplasia. In any case this could be a result of eithuer clonal expansion of cells expressing a particular 'normal' PNA binding phenotype or expression of an 'abnormal' phenotype at the cellular level. Secondly, we were interested in the possible relationship between the expression of PNA reactive glycoconjugates and growth rate, tumorigenicity, metastatic potential and other biological characteristics of transformed mammary cells. Instead of examining paired normal *vs* malignant or metastatic *vs* non-metastatic cell types, we decided to analyse the full spectrum of progression from normal mammary epithelium to benign hyperplasia to neoplasia including resulting tumour cell heterogeneity. In this study we compared murine mammary cells at different stages of progression with respect to PNA binding intensity to the cell surface using flow cytometry and analysed expression of PNA reactive glycoproteins by SDS-PAGE.

Correspondence: F.R. Miller.
Received 10 July 1991; and in revised form 19 November 1991.

### Materials and methods

### Tumours

The preneoplastic mammary hyperplastic alveolar nodule line D2HAN (Medina, 1973) was the source of several tumours (i.e., D2 tumours). EMT6R08 is a long established tumour line derived from a D2 tumour (Loeffler *et al.*, 1990; Rockwell *et al.*, 1972), and D2A1 and D2F2 are clones of a single spontaneous D2 tumour (Mahoney et al., 1985). Polyclonal cell populations derived from new spontaneous D2 tumours arising from D2HAN-filled mammary fatpads were established and maintained as primary or low passage cultures (D2ST1, D2ST2, D2ST3, D2ST4 and D2ST5). Each spontaneous D2 tumour arose in a separate animal except D2ST2 and D2ST3 which came from one animal bearing two tumours in D2HAN implanted fatpads on contralateral sides. D2.0R is one of several clones derived from D2ST5. It was selected for further studies because of its high PNA binding capacity. The D2.0R clone was injected s.c. and, although it was poorly tumorigenic, one of the few resulting tumours was re-established in culture and cloned; D2.1 is one of those randomly chosen clones. The *in vivo* passage of D2.0R cells was performed in two more independent experiments each time yielding a low PNA binding population (clonal or polyclonal) which in one of those experiments was retested for tumorigenicity in syngeneic mice and was found similar to highly tumorigenic D2.1 clone.

A series of sister cell populations derived from a single spontaneous BALB/cfC3H mouse mammary tumour (Dexter et al., 1978; Miller et al., 1986), lines 66c14, 67, 68H, 4T07, 44FTO, and 168FARN, express different phenotypes with respect to *in vivo* and *in vitro* growth and metastasis. Line 64PT is a hybrid of 4TO7 and 68H (Miller et al., 1988b). SP1(M1M1) is a metastatic variant of the SP1 spontaneous CBA/J mammary adenocarcinoma (Elliott et al., 1988) obtained from Dr Bruce Elliott, Queens University, Kingston, Ontario.

Tumour cells were grown in DME supplemented with 5% fetal bovine serum (FBS), 5% calf serum (CS), 2 mM glutamine, 100 U $ml^{-1}$ penicillin, 100 $\mu g\ ml^{-1}$ streptomycin and 1 mM of mixed nonessential amino acids. Culturing of tumour cells in media used for mammary epithelium and D2HAN (see below) did not alter any of their properties.

*Primary cell culture*

Tumours were dissociated mechanically and by treatment with Collagenase type 3 (Worthington) 2 mg $ml^{-1}$ and Hyaluronidase (Sigma) 1 mg $ml^{-1}$ for 1 h on ice with subsequent repetitive agitation for 30 s in a Stomacher Blender (Miller et al., 1990). Normal mammary cell cultures were prepared using the procedure described elsewhere (Miller & McInerney, 1988). Briefly, mammary glands of midpregnant BALB/c mice were aseptically removed, minced, and digested with an enzyme cocktail containing Collagenase type III and hyaluronidase. The digest was then separated by double sedimentation in Hank's Balanced Salt Solution (HBSS) followed by 20 *g* centrifugation on FBS cushion for 1 min. The aggregates (containing mostly epithelial cells) and single cells (mostly stromal cells) were depleted of macrophages by adhesion to plastic for 1 h at 37°C and then plated for 7–10 days in medium. In some experiments stromal and epithelial cells were plated unseparated. Cells were maintained in DME-10 containing 10% NCTC 109 medium (M.A. Bioproducts, Walkersville, MD), 20 mM glucose, 8 $\mu g\ ml^{-1}$ bovine crystallin insulin, and 1 mM oxaloacetic acid. Single cell suspensions were prepared by conventional, brief treatment with 0.25% trypsin in 0.05% EDTA.

*Cell injection*

Animals were injected with 0.2 ml of a single cell suspension in HBSS (calcium and magnesium free). Subcutaneous (s.c.) tumour growth was monitored by periodic measurements of the length (a) and width (b) of tumour and subsequent calculation of tumour weight according to the formula: weight = $a \times b^2/2$. Tumour weight doubling time was estimated by computerised exponential regression and calculation according to the formula: doubling time = ln2/k (k-growth constant) (Rak et al., 1988). The number of lung colonies which developed after intravenous (i.v.) cell injection was determined at necropsy by counting the foci visible under a dissecting microscope on the surface of Bouin fixative-treated lungs.

*In vitro* growth rates were determined by plating $1 \times 10^6$ cells into 60 mm tissue culture dishes and periodically harvesting and counting cells from three randomly chosen dishes. Population doubling times were calculated by regression analysis. .

*Flow cytometry*

The procedulre was described previously (Rak et al., 1991). Briefly, cells were harvested with trypsin-EDTA solution, incubated in DME-10FBS for 1 h on ice, washed, pelleted, and incubated for 40 min with 10–50 $\mu g\ ml^{-1}$ (usually 25 $\mu g\ ml^{-1}$) of fluorescent Peanut lectin (PNA-FITC), Soy bean agglutinin (SBA), or *Griffonia simplicifolia* isolectin (GSIB4) in the presence (control) or absence of an appropriate blocking sugar (galactose, N-Acetylo- galactosamine, or alpha-methyl galactopyranoside, respectively). All the lectin blocking sugars were purchased from Sigma Chemical Co. (St Louis, MO). In some experiments, prior to incubation with lectins, cells were treated with 0.1 U $ml^{-1}$ neuraminidase (type V, *Cl. perfringens*, Sigma Chemical Co., St Louis, MO). After several washes, cells were fixed in 0.1% paraformaldehyde and analysed by FACStar flow cytometer under 420 nm of Innova 90 Argon laser light (Becton Dickinson, Mountain View, CA). Mean specific fluorescence intensity was calculated by subtraction of the mean fluorescence intensity of cells incubated with sugar-inactivated lectin from the mean fluorescence of cells stained with intact lectin. Significance of differences was confirmed at the confidence level of $P < 0.001$ according to Kolmogorow-Smirnow statistics. Some variability in modal fluorescence between different stainings of the same cell population was seen. This was a result of different batches and, in some cases, concentrations of the fluorescent lectins used in individual experiments. Also setting of the instrument for each individual analysis might have been a source of some variability of the numerical values describing fluorescence intensity. Parallel fluctuations of those values were seen in control samples and between different cell types, so they did not affect the results. We also noticed that in the case of clonal D2 tumour cell lines untreated with neuraminidase (D2.0R, D2.1, D2A1) the staining intensity is up to 30% lower when the cells remain confluent as compared to the cells in the log phase of growth. Although the cells were always analysed at approximately the same (70–90%) confluency some fluorescence fluctuations may be due to this phenomenon. The latter, however, did not obscure several fold relative differences between the cell lines. Also, size and direction of the confluency dependent shift was similar for all of the three clonal D2 tumour cell lines. The time course and nature of this confluence-related shift in expression of PNA binding sites was not examined in detail. Double staining with propidium iodide and PNA-FITC did not suggest any S-phase specific expression of the lectin binding sites for any of these lines.

*PNA overlays*

Semiconfluent cell cultures were harvested with 2 mM EDTA, washed in PBS, pelleted, and treated with lysis buffer containing 0.5% NP-40, 1 mM EDTA, and 1 mM phenylmethylsulfonylfluoride in PBS. The protein equivalent of $3 \times 10^6$ cells was separated by SDS-PAGE in 8% gel under reducing conditions, transferred to a nitrocellulose filter, and treated (or not) with 1 mU $ml^{-1}$ neuraminidase type V and with 0.25 $\mu g\ ml^{-1}$ of PNA. The lectin binding protein was visualised by autoradiography after incubating the filter with anti-PNA rabbit antibody (Accurate Chemicals and Scientific Corporation, Westbury, NY) followed by incubation in anti-rabbit $I^{125}$-labelled antibody (Amersham).

### Results

The cultured cells from normal mammary gland stained strongly and specifically with PNA-FITC (Figure 1). Populations enriched for epithelial or stromal component displayed respectively high and low PNA binding (Figure 2). Staining with Soybean agglutinin (SBA) or *Griffonia simplicifolia* isolectin (GSIB4) did not reveal any difference between epithelial and stromal mammary components (not shown).

A gradual decrease in PNA binding (Figure 3a and 3b) but not in SBA binding (Figure 3c) accompanied progression from preneoplastic D2HAN lesions to spontaneous D2HAN derived tumours and highly malignant clonal cell lines D2A1 and D2F2. Histogram overlaps in Figure 3 suggest a great deal of variability and perhaps the presence of high and low PNA binding subpopulations within both preneoplastic D2HAN lesions and tumours. By isolating and screening several clones from a spontaneous D2 tumour, one clonal population (D2.0R) was obtained which stained very strongly with PNA-FITC (Figure 4). From a tumour formed after injecting $1 \times 10^6$ D2.0R cells s.c., a primary tissue culture was established. D2.1 is representative of eight clones obtained from that culture. The ability of clone D2.1 to bind PNA was intermediate to the parental D2.0R clone and the highly malignant D2A1 tumour line (Figure 4). Having a panel of those three clonal D2HAN derived tumour cell lines expressing high, intermediate and low PNA binding ability, we began to compare their malignant properties. Clones D2A1 (low -) and D2.1 (intermediate PNA binding) were able to generate 100% incidence of s.c. tumours when as few as $5 \times 10^4$ cells were injected, whereas D2.0R (high PNA binding) failed to initiate tumours in those experiments and initiated tumours in less than 100% animals after injection of $5 \times 10^5$ cells (not shown). The differential growth kinetics of these lines after s.c. injection of $1 \times 10^6$ cells is shown in Figure 5. All three

**Figure 1** PNA-FITC binding profiles of normal mammary cells (MAM) stained with 50μg ml$^{-1}$ of intact (MAM-PNA) or 0.2 M galactose inactivated (MAM-GAL) lectin.

**Figure 2** PNA-FITC (25 μg ml$^{-1}$) binding profiles of epithelial and stromal cells isolated from normal mammary glands.

**Figure 3** PNA-FITC binding profiles of normal mammary cells (MAM), preneoplastic (D2HAN tissue), tumour derived (D2HAN tumour) and malignant D2HAN related clones (D2A1, D2F2) – two experiments are shown in panels **a** and **b**. SBA-FITC staining of normal mammary cells (MAM) and three D2HAN related tumour cell populations – panel **c**.

**Figure 4** PNA-FITC binding profiles of three D2HAN derived tumour cell clones: D2A1 (——), D2.1 (·····), and D2.0R (----). Staining of D2.0R was significantly greater ($P<0.001$) than either D2.1 or D2A1.

**Figure 5** Tumour growth of three D2HAN derived tumour cell clones: D2A1 (□), D2.1 (●), D2.0R (■). For each tumour line 5–6 syngeneic BALB/c mice were injected s.c. with $1 \times 10^6$ cells.

**Figure 6** Lung colonisation potential of three D2HAN derived tumour cell clones after i.v. injection of $5 \times 10^5$ cells.

lines produced tumours when $1 \times 10^6$ cells were injected s.c. Line D2A1 had the shortest latency and a faster growth rate (doubling time of 8 days) than either D2.1 or D2.0R. High PNA binding line D2.0R had a longer latency than line D2.1 but the growth rate of the two was similar once palpable. The ability to form lung colonies after i.v. injection of $5 \times 10^5$ was greatest for D2A1 cells (Figure 6). All the mice developed visible nodules by the time of necropsy at 28 days. The number of lung colonies per lung ranged between 11 and 38 (median 19). Furthermore, extrapulmonary foci in liver, kidney, eye, and subcutis were found in 67% of animals. After injection of D2.1 or D2.0R cells, single lung colonies appeared in some mice by the time of necropsy (72 days). No extrapulmonary deposits were found. Neither could we find spontaneous metastases after s.c. injection of those two cell lines; however, spontaneous metastases were observed in mice bearing s.c. D2A1 tumours (not shown). Despite differences described above, the three D2HAN derived tumour cell population had similar *in vitro* growth potential. Doubling time of D2A1 line ($0.68 \pm 0.15$ days) was slightly shorter than that for D2.1 or D2.0R cells ($0.76 \pm 0.10$ and $0.75 \pm 0.10$ days, respectively).

To determine the kinetics of the loss of PNA-binding capacity, tumours were initiated by s.c. injection of $1 \times 10^6$ D2.0R cells. The two largest of the early appearing tumours were removed after 67 days of growth (tumour weights 108 and 600 mg) and cells cultured. Later, on day 103, two large (5235 mg and 3179 mg) and two smaller tumours (847 mg and 196 mg) were used to establish primary tissue cultures. After 3–4 passages *in vitro* the PNA binding abilities of these cell populations were compared to the parental D2.0R clone (Figure 7). The lectin reactivity of cells derived from the large tumours was substantially (four times) lower than that of the parental line ($P<0.001$). Cells derived from large tumours were also more tumorigenic after injection of $1 \times 10^6$ cells s.c. than parental D2.0R cells (not shown). Small and early tumour-derived cells displayed intermediate values suggesting that the process of losing the expression of PNA binding sites is gradual and time dependent. Only *in vivo* passage was able to cause that effect since the D2.0R clone maintained its PNA-binding phenotype for several months in culture (not shown).

Differential sialylation of the lectin binding sites apparently plays a role in these phenotypic changes because neuraminidase treatment was able to abrogate the quantitative differences (not shown). Qualitative analysis of the pattern of PNA binding glycoproteins (Figure 8) revealed the presence of a highly PNA-reactive high molecular weight band (approximately 150 Kd) in lysates of parental clone D2.0R cells which is missing in lysates of the tumour derived cells including clone D2.1 obtained from D2.0R tumour. Desialylation of that material revealed virtually identical patterns

**Figure 7** PNA-FITC binding to clone D2.0R, its subclone D2.1, and uncloned populations derived from tumours formed after injecting $1 \times 10^6$ D2.0R cells s.c. (early tumour, ET; small late tumour, Sm; and large late tumour, Lg). Means represent three experiments of D2.0R, D2.1, ET, and Lg, but Sm was analysed only twice. Staining of D2.0R cells was significantly greater than the staining of ET ($P<0.05$), Sm ($P<0.01$), Lg ($P<0.01$), or D2.1 ($P<0.001$). Staining of ET was significantly greater than Sm, Lg, or D2.1 ($P<0.001$).

**Figure 8** PNA reactive glycoproteins in whole cell lysates of clone D2.0R, subclone D2.1, and uncloned populations derived from tumours formed by injecting D2.0R cells s.c.

consisting of four PNA reactive common bands (220 kd, 180 kd, 150 kd and 85 kd) for both parental D2.0R cells and tumour derived cells. This suggests that specific sialylation of gp 150 contributes to the differences observed.

Several established mammary tumour cell lines displayed rather weak PNA binding capacities (Table I). Although some variability among them was found, no general correlation between PNA staining intensity and expression of known biological characteristics such as growth and metastatic potentials or drug resistance was found. With the exception of the poorly tumorigenic clone D2.0R, cells from D2 series of premalignant HAN lesions bound less PNA-FITC than cells from normal mammary gland and from D2HAN preneoplastic lesions (Figure 4, Table I). This includes both established cell lines (D2A1, D2F2, EMT6RO8) and primary cultures of D2HAN derived tumours (Table I). Because pretreatment with neuraminidase abolishes the differential binding of PNA by normal and transformed mammary cells (Table I), differential sialylation rather than an absolute absence of PNA-specific oligosaccharides on the cell surface may be responsible. In many cases, however, the expression of tumorigenic potential seems to be associated with low PNA binding to mammary cells.

A single PNA reactive band (220 kd) common for normal, premalignant, and malignant mammary cells was observed (Figure 9). Regardless of neuraminidase treatment, lysates of D2HAN derived tumours and BALB/cfC3H tumours did not exhibit the very large molecular weight band (>250 kd) seen in D2HAN and normal mammary epithelium (arrow, Figure 9). A major band of approximately 90 kd was present in lysates of normal mammary epithelium, but not in D2HAN or any tumour cell types. A band of this size was present in some tumour cell lysates following neuraminidase treatment. A major band of approximately 150 kd was detected in lysates of normal mammary epithelium and to a lesser extent in preneoplastic D2HAN cells. This band was detected in poorly tumorigenic D2.0R cells and almost completely disappeared from the material obtained from D2A1 malignant cells. This band was detected in other tumour lysates after neuraminidase treatment.

### Discussion

We were struck by the observation that PNA binding to neoplastic mammary cells was dramatically reduced in comparison to their normal epithelial counterparts. Neuraminidase treatment resulted in abrogation of that difference indicating that masking of the lectin binding sites by sialic acid was involved. A similar difference was observed in three unrelated panels of mammary tumour cell populations derived from spontaneous tumours which arose in three different strains of syngeneic mice.

It is well documented that abnormal expression of carbohydrates correlates with neoplasia (Feizi, 1985*a*); Hakomori, 1989). Roles for both N-linked (Dennis & Laferte, 1989 and references therein) and 0-linked (Schirrmacher, 1982) oligosaccharides in invasion and metastasis have been postulated. Oncodevelopmental antigens have been detected in breast cancer specimens using monoclonal antibodies specific for blood group related carbohydrates (Feizi, 1985*a*; Feizi, 1985*b*; Gooi, 1985*a*; Gooi, 1985*b*) and PNA (Springer, 1984). Sialylation of various glycoconjugates correlates with metastatic phenotype in different systems (Benedetto *et al*., 1989; Bresalier *et al*., 1990; Corfield *et al*., 1990; Friedman *et al*., 1988; Passaniti & Hart, 1988; Dennis *et al*., 1989). However, there is also evidence indicating that in some systems the expression of non-sialylated structures binding SBA (Buckey *et al*., 1988) or PNA (Badenoch-Jones *et al*., 1987; Schirrmacher *et al*., 1982) is associated with metastasis. Sialylation of surface carbohydrates was alternatively correlated with cell proliferation (Kinoshita *et al*., 1989) or differentiation (Kinoshita *et al*., 1989; Kuratsu *et al*., 1990). In some reports only subtle changes in glyconjugate expression patterns were seen

**Table I** Flow cytometry analysis of PNA-FITC binding to mouse mammary tumour cells

| *Tumour cell population* | *Mean relative fluorescence intensity (% normal)* (−)Neuraminidase | *Neuraminidase treated* | *Characteristics* TUM | *LCF* | *MET* |
|---|---|---|---|---|---|
| Clonal cell lines established from mammary tumours derived from D2HAN lesions | | | | | |
| D2.0R | 183,388 | 133,183 | +/− | − | − |
| D2.1 | 36,44,51,53,53,67 | 148 | + | − | − |
| D2A1 | 4,7,9,12,23 | 74,80,124 | +++ | +++ | + |
| D2F2 | 8 | 87 | +++ | ND | ND |
| EMT6R08 | 14 | ND | ++ | ND | ND |
| Polyclonal cell populations isolated from D2HAN derived mammary tumours (passage #1–5) | | | | | |
| D2ST1 | 25,36 | 156 | NT | NT | − |
| D2ST2 | 45 | 172 | NT | NT | − |
| D2ST3 | 22 | 138 | NT | NT | − |
| D2ST4 | 29 | ND | NT | NT | NT |
| Clonal subpopulations derived from a single spontaneous mammary tumour in BALB/cfC3H mouse | | | | | |
| 67 | 1,2 | ND | ++ | − | − |
| 66c14 | 2,2 | 57 | +++ | +++ | +++ |
| 168FARN | 19 | 114 | +++ | +++ | − |
| 4T07 | 1,2 | 91 | +++ | ++++ | +/− |
| 68H | 4 | 85 | +/− | ND | ND |
| 64pT | 3,7,12 | 99 | ++ | ++ | + |
| Subpopulation of the spontaneous mammary carcinoma in CBA/J mouse | | | | | |
| SP1(M1M1) | 30 | ND | +++ | ND | ND |

Mean specific fluorescence intensity was calculated by subtraction of the mean fluorescence intensity of cells incubated with galactose-inactivated lectin from the mean fluorescence of cells stained with intact lectin. Mean relative fluorescence intensity is the percent of the mean specific fluorescence intensity of mouse mammary epithelial cells. Each value represents an individual experiment. TUM – tumorigenicity after s.c. inoculation; LCF – lung colony formation after i.v. injection of tumour cells; MET – spontaneous metastasis in distant organs detected at autopsy of the primary or secondary tumour bearer; ND – not done; NT – no transplantation to a secondary bearer was performed.

**Figure 9** PNA reactivity of SDS-PAGE separated glycoproteins from whole cell lysates isolated from neoplastic (lanes 1–7), preneoplastic (lane 8), and normal (lane 9) mammary cell populations with or without neuraminidase treatment.

between more and less malignant cell lines (Steck & Nicolson, 1983; Tresser & Nicholson, 1988). Some studies suggest that sugar structures are directly involved in determination of cell phenotype due to modification of physical and/or biochemical properties of their surfaces (Dennis & Laferte, 1989; Friedman *et al.*, 1988; Schirrmacher *et al.*, 1982). The importance of a particular structure seems to depend on the genetic background of cells expressing it. For example PNA receptor is normally expressed by human gastric mucosa of so called 'non-secretors' but is considered to be a tumour associated feature in 'secretors' (Feizi, 1985*b*). Species specificity was also reported (Galili & Machmer, 1989). On the other hand specific functions of some adhesion molecules, their ligands and other important known glycoconjugates were shown to be regulated by their glycosylation (Diamond *et al.*, 1991; Larsen *et al.*, 1990; Oz *et al.*, 1989; Rabinovitz *et al.*, 1991). Depite the plethora of information, which was only exemplified above, the clue for understanding the role of aberrant glycosylation in tumour progression seems still to be missing. The most common strategy in this area of research has been an analysis of paired cell populations usually representing a rather narrow 'window' of the whole process, for example a transition from low to high metastatic phenotype. We felt that because of the stepwise nature of tumour progression at the genetic level (Chen *et al.*, 1989 and references therein) glycosylation changes may also follow a sequential course. This implies that initial conditions (differentiated phenotype) might be important. The expression of differentiation related carbohydrates recognisable by lectins was reported for several systems including hematopoietic cells (Reimann *et al.*, 1984), endothelium (Alroy *et al.*, 1987) and mammary epithelium (Rudland, 1987). The latter comprises several phenotypically distinct cell populations (Rudland, 1987; Sonnenberg *et al.*, 1986). Interestingly, in rat and humans the main populations can be classified on the basis of their PNA binding patterns (Rudland, 1987; Newman *et al.*, 1979). In these studies ductal and alveolar cells bound the lectin with or without neuraminidase treatment, respectively. Myoepithelial and stromal cells were negative regardless of neuraminidase treatment. In the murine system we observed PNA binding sites widely distributed amongst mammary cell populations in culture (see Figure 1). Flow cytometry, however, clearly showed 20-fold lower PNA binding to stromal cells than to mammary epithelium (compare Figure 2) thus indicating the quantitative nature of this differentiation marker. Further analysis revealed sequential quantitative and qualitative changes in PNA binding pattern during development of preneoplastic hyperplastic (D2HAN) and subsequent progression of the neoplasia. (i) We observed a gradual decrease in expression of PNA binding sites throughout the whole process. No such general trend was seen in binding of SBA, GSIB4, or L-PHA (Rak, Miller, unpublished). (ii) We observed an inverse correlation of PNA binding capacity and the expression of the malignant phenotype (tumorigenicity, metastatic potential) among D2HAN related cells seen in flow cytometry and preliminary clonal analysis suggest that most of the PNA binding sites are being lost during transition from the premalignant to tumorigenic state probably due to progressive cellular alterations rather than selection of pre-existing populations within D2HAN lesion (compared phenotypes of related clones D2.0R and D2.1). There are metastatic and nonmetastatic populations amongst the low PNA binding tumour cell lines derived from BALB/cfC3H mouse mammary tumour (see Table I) but we have not observed a metastatic and at the same time highly PNA binding mammary tumour. (iii) There was a loss of high molecular weight (conceivably mucin-like) PNA reactive glycoproteins (>250 kd) during transition from hyperplasia to neoplasia; (iv) loss or hypersialylation of 90 kd glycoprotein during development of neoplasia; and (v) hypersialylation of 150 kd glycoprotein in the case of highly tumorigenic but not in normal, preneoplastic and poorly tumorigenic D2.0R mammary cells. Recognition of these alterations leads to several further questions. First, do they represent consequences (markers) of tumour progression or are some of them necessary components of the malignant phenotype. Second, which component of a particular change is essential; the carbohydrate structure or the whole glycoprotein? Structural analysis of carbohydrates recognised by PNA (T-antigen and other terminal galactose-rich O-linked structures) can be analysed by using a panel of monoclonal antibodies (Feizi, 1985; Hakomori, 1989). We also intend to screen our material with antibodies recognising known molecules of corresponding size potentially involved in tumour progression. Mammary mucins (Taylor-Papadimitriou *et al.*, 1990; McKenzie *et al.*, 1990), E-cadherins (Nagafuchi *et al.*, 1987), CD44 (Brown *et al.*, 1991) or betal integrin (Oz *et al.*, 1989) are some of the obvious candidates. There is also an interesting size similarity between a sialylated gp 150 involved in liver metastasis formation by murine leukaemia cells (Benedetto *et al.*, 1989) and the 150 kd glycoprotein sialylated during *in vivo* passage of mammary D2.0R tumour cells. Sialylation of the latter molecule appeared to be involved in full expression (or derepression?) of tumorigenicity. Interestingly, the highly tumorigeneic subclone (D2.1) and polyclonal populations (ET, Lg, Sm), all expressing the sialylated form of this glycoprotein (see Figure 7), seem to be generated during interaction of the parental D2.0R clone with host environment since hypersialylation of that molecule occurred only after *in vivo* passage, but not during several months in culture. It is premature to speculate on mechanisms of this phenotypic switch *in vivo* until they can be reproduced by relevant *in vitro* systems. Both selection against high PNA binding variants and induction of the low PNA binding phenotype are conceivable. We are currently exploring a number of possibilities including tumour cell interaction with stromal cells, with extracellular matrix, and with the immune system.

Thus, we have been able to demonstrate a series of sequential quantitative and qualitative changes in expression of PNA reactive glycoconjugates during murine mammary tumour development and progression. Further studies on the nature of those alterations and identity of molecules involved are currently being pursued. Hypersialylation of a 150 kd glycoprotein seems to be important for host-dependent modulation of the tumorigenic phenotype of transformed mammary cells.

Supported by USPHS Grant CA 28366 from the National Cancer Institute. We thank Drs Gloria Heppner, Avraham Raz, Wei-Zen Wei, Stuart Ratner, and Jane Tsai, for their great intellectual partnership in these studies. The constant friendly and professional help of Mrs Margaret Peterson was a great support for us.

### References

ALROY, J., GOYAL, V. & SKUTELSKY, E. (1987). Lectin histochemistry of mammalian endothelium. *Histochemistry*, **86**, 603.

BADENOCH-JONES, P., CLAUDIANOS, C. & RAMSHAW, I.A. (1987). Lectin-binding characteristics of related high- and low-metastatic rat mammary adenocarcinoma cell lines. *Invasion Metastasis*, **7**, 284.

BENEDETTO, A., ELIA, G., SALA, A. & BELARDELLI, F. (1989). Hyposialylation of high-molecular-weight glycoproteins parallels the loss of metastatic potential in wheat-germ agglutinin-resistant Friend leukemia cells. *Int. J. Cancer*, **43**, 126.

BOLSHER, J.G.M., VAN DER BIJL, M.M.W., NEEFHES, J.J., HAAL, A., SMETS, L.A. & PLOEGH, H.L. (1988). Ras(proto)oncogene induced N-linked carbohydrate modification: temporal relationship with induction of invasive potential. *EMBO J.*, **7**, 3361.

BRESALIER, R.S., ROCKWELL, R.W., DAHIYA, R., DUH, Q.-Y. & KIM, Y.S. (1990). Cell surface sialoprotein alterations in metastatic murine colon cancer cell lines in an animal model for colon cancer metastasis. *Cancer Res.*, **50**, 1299.

BROWN, T.A., BOUCHARD, T., ST. JOHN, T., WAYNER, E. & CARTER, W.G. (1991). Human keratinocytes express a new CD44 core protein (CD44E) as a heparan-sulfate intrinsic membrane proteoglycan with additional exons. *J. Cell Biol.*, **113**, 207.

BUCKLEY, N.D. & CARLSEN, S.A. (1988). Involvement of soybean agglutinin binding cells in the lyphatic metastasis of the R3230AC rat mammary adenocarcinoma. *Cancer Res.*, **48**, 1451.

CHEN, L.-C., DOLLBAUM, C. & SMITH, H.S. (1989). Loss of heterozygosity on chromosome 1q in human breast cancer. *Proc. Natl Acad. Sci. USA*, **86**, 7204.

CORFIELD, A.P., CLAMP, J.R., CASEY, A.D. & PARASKEVA, C. (1990). Characterization of sialic- acid-rich mucus glycoprotein secreted by a premalignant human colorectal adenoma cell line. *Int. J. Cancer*, **46**, 1990.

DENNIS, J.W. & LAFERTE, S. (1989). Importance of cell surface carbohydrates in tumor cell metastasis. In *Cancer Metastasis: molecular and cellular biology, host immune responses, and perspective for treatment*. Schirrmacher, V. & Schwartz-Albiez, R. (eds), p. 86. Springer-Verlag: Berlin, New York.

DEXTER, D., KOWALSKI, H., BLAZER, B., FLIEGEL, Z., VOGEL, R. & HEPPNER, G. (1978). Heterogeneity of tumor cells from a single mouse mammary tumor. *Cancer Res.*, **38**, 3174.

DIAMOND, M.S., STAUNTON, D.E., MARLIN, S.D. & SPRINGER, T.A. (1991). Binding of the integrin Mac-1 (CD11b/CD18) to the third immunoglobulin-like domain of ICAM-1 (CD54) and its regulation by glycosylation. *Cell*, **65**, 961.

ELLIOTT, B.E., ARNOLD, M.M., WEI, W.-Z. & MILLER, F.R. (1988). Expression of epithelial-like markers and class I major histocompatibility antigens by a murine carcinoma growing in the mammary gland and in metastases: orthotopic site effect. *Cancer Res.*, **48**, 7237.

FEIZI, T. (1985*a*). Carbohydrate antigens and human cancer. *Cancer Surv.*, **4**, 245.

FEIZI, T. (1985*b*). Demonstration by monoclonal antibodies that carbohydrate structures of glycoproteins and glycolipids are onco-developmental antigens. *Nature*, **314**, 53.

FRANKLIN, W.A. (1983). Tissue binding of lectins in disorders of the breast. *Cancer*, **51**, 295.

FRIEDMAN, J., LEVINSKY, H., ALLALOUF, D. & STAROSELSKY, A. (1988). Sialic acid content in mouse myeloma cells and derived B-cell hybridomas with different metastatic potentials. *Cancer Lett*, **43**, 79.

GALILI, U. & MACHER, B.A. (1989). Interaction between anti-Gal and human tumor cells: a natural defense mechanism? *J. Natl Cancer Inst.*, **81**, 178.

GOOI, H.C., JONES, N.J., HOUNSELL, E.F., SCUDDER, P., HILKENS, J., HILGERS, J. & FEIZI, T. (1985*a*). Novel antigenic specificity involving the blood group antigen Le(a) in combination with onco-development antigen SSEA-1, recognized by two monoclonal antibodies to human milk fat globule membranes. *Biochem. Biophys. Res. Comm.*, **131**, 543.

GOOI, H.C., JONES, N.J., HILKENS, J., HILGERS, J. & FEIZI, T. (1985*b*). Lewis blood-group related specificities of monoclonal antibodies designated MAM-3a, b, c against human milk-fat globule membranes. *Glycoconjugate J.*, **2**, 409.

HAKOMORI, S.-I. (1989). Aberrant glycosylation in tumors and tumor-associated carbohydrate antigens. *Adv. Cancer Res.*, **52**, 257.

HOWARD, D.R., FERGUSON, P. & BATSAKIS, J.G. (1981). Carcinoma associated cytostructural antigenic alterations: detection by lectin binding. *Cancer*, **47**, 2872.

KINOSHITA, Y., SATO, S. & TAKEUCHI, T. (1989). Cellular sialic acid level and phenotypic expression in B16 melanoma cells: comparison of spontaneous variations and bromodeoxyuridine- and theophylline-induced changes. *Cell Struct. Funct.*, **14**, 35.

KURATSU, J. SUEYOSHI, N., MIHARA, Y. & USHIO, Y. (1990). Localization and significance of peanut agglutinin binding sites on ependymoma cells. *Acta Neuropathologica*, **79**, 634.

LARSEN, E., PALABRICA, T., SAJER, S., GILBERT, G.E., WAGNER, D.D., FURIE, B.C. & FURIE, B. (1990). PADGEM-dependent adhesion of platelets and neutrophils is mediated by a lineage specific carbohydrate, LNF III (CD15). *Cell*, **63**, 467.

LOEFFLER, D.A., KENG, P.C., BAGGS, R.B. & LORD, E.M. (1990). Lymphocytic infiltration and cytotoxicity under hypoxic conditions in the EMT6 mouse mammary tumor. *Int. J. Cancer*, **45**, 462.

MAHONEY, K.H., MILLER, B.E. & HEPPNER, G.H. (1985). FACS quantitation of leucine aminopeptidase and acid phosphatase on tumor associated macrophages from metastatic and nonmetastatic mouse mammary tumors. *J. Leukoc. Biol.*, **38**, 573.

MCKENZIE, I.F.C. & XING, P.-X. (1990). Mucins in breast cancer: recent immunological advances. *Cancer Cells*, **2**, 75.

MEDINA, D. (1973). Preneoplastic lesions in mouse mammary tumorigenesis. *Meth. Cancer Res.*, **7**, 3.

MILLER, F.R. & MCINERNEY, D. (1988). Epithelial component of host-tumor interaction in the orthotopic site preference of mouse mammary tumor. *Cancer Res.*, **48**, 3698.

MILLER, F.R., MCINERNEY, D., ROGERS, C. & MILLER, B.E. (1988*b*). Spontaneous fusion between metastatic mammary tumour subpopulations. *J. Cell Biochem.*, **36**, 129.

MILLER, B.E., ASLAKSON, C.J. & MILLER, F.R. (1990). Efficient recovery of clonogenic stem cells from solid tumors and occult metastatic deposits. *Invasion Metastasis*, **10**, 101.

MULLER-HOLZNER, E., MARTH, C., KOFLER, E., DAXENBICHLER, G. & HOFSTADTER, F. (1985). Lectin binding sites in cultured human breast cells. *Breast Cancer Res. Treat.*, **5**, 277.

NAGAFUCHI, A., SHIRAYOSHI, Y., OKAZAKI, K., YASUDA, K. & TAKEICHI, M. (1987). Transformation of cell adhesion properties by exogenously introduced E-cadherin cDNA. *Nature*, **329**, 341.

NEWMAN, R.A., KLEIN, P.J. & RUDLAND, P.S. (1979). Binding of peanut lectin to breast epithelium, human carcinoma and cultured rat mammary stem cell: use of the lectin as a marker of mammary differentiation. *J. Natl Cancer Inst.*, **68**, 1339.

OZ, O.K., CAMPBELL, A. & TAO, T.-W. (1989). Reduced cell adhesion to fibronectin and laminin is associated with altered glycosylation of beta1 integrins in a weakly metastatic glycosylation mutant. *Int. J. Cancer*, **44**, 343.

PASSANITI, A. & HART, G.W. (1988). Cell surface sialylation and tumor metastasis. Metastatic potential of B16 melanoma variants correlates with their relative numbers of specific penultimate oligosaccharide structures. *J. Biol. Chem.*, **263**, 7591.

RABINOWITZ, S.S. & GORDON, S. (1991). Macrosialin, a macrophage-restricted membrane sialoprotein differentially glycosylated in response to inflammatory stimuli. *J. Exp. Med.*, **174**, 827.

RAK, J., KUSNIERCZYK, H., STRAZADALA, L. & RADZIDOWSKI, C. (1988). Transplantable mouse 16/c mammary adenocarcinoma as a model in experimental cancer therapy. I. Kinetics of growth and spread. *Arch. Immun. Ther. Exp.*, **35**, 325.

RAK, J.W., BASOLO, F., ELLIOTT, J.W., RUSSO, J. & MILLER, F.R. (1991). Cell surface glycosylation changes accompaning immortalization and transformation of normal human mammary epithelial cells. *Cancer Lett.*, **57**, 27.

RAZ, A., PAZERINI, G. & CARMI, P. (1989). Identification of the metastasis associated, galactoside binding lectin as a chimeric gene product with homology to IgE-binding protein. *Cancer Res.*, **49**, 3489.

REIMANN, J., EHMAN, D. & MILLER, R.G. (1984). Differential binding of lectins to lymphopoetic and myelopoetic cells in murine marrow as revealed by flow cytometry. *Cytometry*, **5**, 194.

ROCKWELL, S.C., KALLMAN, R.F. & FAJARDO, L.F. (1972). Characteristics of a serially transplanted mouse mammary tumor and its tissue-culture-adopted derivative. *J. Natl Cancer Inst.*, **49**, 735.

RUDLAND, P.S. (1987). Stem cells and the development of mammary cancers in experimental rats and in humans. *Cancer Metastasis Rev.*, **6**, 55.

RUDLAND, P.S., HUGHES, C.M., FERNS, S.A. & WARBURTON, M.J. (1989). Characterization of human mammary cell types in primary culture: immunofluorescent and immunocytochemical indicators of cellular heterogeneity. *In Vitro Cell. Dev. Biol.*, **25**, 23.

RUSSO, J., REINA, D., FREDERICK, J. & RUSSO, I. (1988). Expression of phenotypical changes by human breast epithelial cells treated with carcinogens *in vitro*. *Cancer Res.*, **48**, 2837.

SCHIRRMACHER, V., ALTEVOGT, P., FOGEL, M. & 13 others (1982). Importance of cell surface carbohydrates in cancer cell adhesion, invasion and metastasis. Does sialic acid direct metastatic behavior. *Invasion Metastasis*, **2**, 313.

SMETS, L.A. & VAN BEEK, W.P. (1984). Carbohydrates of the tumor cell surface. *Biochim. Biophys. Acta.*, **730**, 237.

SONNENBERG, A., DAAMS, H., VAN DER VALK, M.A., HILKENS, J. & HILGERS, J. (1986). Development of mouse mammary gland: identification of stages in differentiation of luminal and myoepithelial cells using monoclonal antibodies and polyvalent antiserum against keratin. *J. Histochem. Cytochem.*, **34**, 1037.

SPRINGER, G.F. (1984). T and Tn, general carcinoma autoantigens. *Science*, **224**, 1198.

STECK, P. & NICOLSON, G. (1983). Cell surface glycoproteins of 13762NF mammary adenocarcinoma clones of differing metastatic potentials. *Exptl. Cell Res.*, **147**, 255.

TAYLOR-PAPADIMITRIOU, J., GENDLER, S.J., BURCHELL, J., LALANI, E.-N. & LANCASTER, C. (1990). Immunogenicity and predicted sequence of a mucine core protein which is aberrantly glycosylated in breast cancer. *Proc. Amer. Assoc. Cancer Res.*, **31**, 468.

TRESSER, R.J. & NICOLSON, G.L. (1988). Cell surface biochemical and metastatic properties of *Lens culinaris* hemagglutinin-binding variants of a murine large cell lymphoma. *Invasion Metastasis*, **8**, 351.

YOGEESWARAN, G. (1983). Cell surface glycolipids and glycoproteins in malignant transformation. *Adv. Cancer Res.*, **38**, 289.